# Predictors of suicide in the patient population admitted to a locked-door psychiatric acute ward

**DOI:** 10.1371/journal.pone.0173958

**Published:** 2017-03-16

**Authors:** Roar Fosse, Wenche Ryberg, Merete Kvalsvik Carlsson, Jan Hammer

**Affiliations:** Department of research and development, Division of mental health and addiction, Vestre Viken hospital trust, Lier, Norway; University of Toronto, CANADA

## Abstract

**Objective:**

No prior study appears to have focused on predictors of suicide in the general patient population admitted to psychiatric acute wards. We used a case-control design to investigate the association between suicide risk factors assessed systematically at admission to a locked-door psychiatric acute ward in Norway and subsequent death by suicide.

**Method:**

From 2008 to 2013, patients were routinely assessed for suicide risk upon admission to the acute ward with a 17-item check list based on recommendations from the Norwegian Directorate of Health and Social Affairs. Among 1976 patients admitted to the ward, 40 patients, 22 men and 18 women, completed suicide within December 2014.

**Results:**

Compared to a matched control group (*n* = 120), after correction for multiple tests, suicide completers scored significantly higher on two items on the check list: presence of suicidal thoughts and wishing to be dead. An additional four items were significant in non-corrected tests: previous suicide attempts, continuity of suicidal thoughts, having a suicide plan, and feelings of hopelessness, indifference, and/or aggression. A brief scale based on these six items was the only variable associated with suicide in multivariate regression analysis, but its predictive value was poor.

**Conclusion:**

Suicide specific ideations may be the most central risk markers for suicide in the general patient population admitted to psychiatric acute wards. However, a low predictive value may question the utility of assessing suicide risk.

## Introduction

Suicide is overrepresented in people with mental illness [1]. The odds for suicide in severe depression, schizophrenia, and bipolar disorder are approximately 3–10 times that of the general population, with a relatively higher increased risk in males than females [2,3]. At the same time, mental illness is a poor predictor since suicide does not occur in 95% to 97% of all cases [2,3]. A focus in the field of suicidology long has been to derive at better criteria for the assessment and formulation of risk in groups of people with mental illness.

The patient population admitted to inpatient treatment in specialized mental health care represents a highly increased suicide risk [4], with studies reporting a 50 to 200 times increased risk as compared to the population at large [5]. In psychiatric inpatients, an array of risk factors for suicide has been identified. In two meta-analyses that together included 42 studies and close to 3500 suicide completers, central suicide risk factors were prior suicide attempts and deliberate self-harm, family history of suicide, suicidal ideation, depression, hopelessness, agitation, and social or relationship problems [6,7]. Single studies have reported additional risk factors, including alcohol and substance abuse, a poor social network and social withdrawal, command hallucinations, delusions, diagnosis of other mental disorders than depression–including bipolar disorder and schizophrenia, coexisting significant physical illness, family history of mental illness, multiple admissions to inpatient treatment, unplanned discharge, and prescription of antidepressants [8–14].

Clinicians are charged with the tasks of assessing and formulating a patient’s risk for suicide in the near future. However, it has been claimed that models for clinicians’ formulation of suicide risk traditionally have been based on intuitive rather than research-based understandings, making suicide assessment and risk formulation as much an art as a science [15]. At the same time, it has been questioned whether identified risk factors have any real heuristic value in inpatient settings. For example, based on their meta-analysis of suicide following discharge from inpatient treatments, Large et al. [6] contended, “risk categorization is of no value in attempts to decrease the number of patients who will complete suicide after discharge” (p. 619). A great deal of research remains to identify suicide predictors, to better inform clinicians how to assess, evaluate, and plan treatment of those at acute and heightened risk [15].

Increased suicide risk (suicidal ideation or plans, nonsuicidal self-injurious behaviors and suicide attempt) may be particularly characteristic among patients admitted to psychiatric acute wards. A study from London reported that risk of self-harm and the need to prevent suicide was the major cause or a contributing cause for 36% of the admissions to acute wards [16]. A study in Norway where suicide risk was assessed prospectively for all patients, indicated that more than half of all first admissions and more than 60% of readmissions to psychiatric acute wards were related to suicide risk [17]. Given its high incidence, research on patients with increased suicide risk at psychiatric acute wards is alarmingly scarce. A reason may be that data on suicidal risk are rarely recorded routinely at acute wards in a way that allows for systematic analysis [17].

### Aims of the study

We had the opportunity to analyze the association between suicide risk factors and subsequent suicide in the general inpatient population at an acute psychiatric ward at Blakstad hospital in Eastern Norway. Late 2007, the ward implemented suicide risk screening as a routine at admission [18], following recommendations in Norwegian national guidelines to systematically assess specific suicide risk factors for all patients in mental health care services [19]. We used a case-control design to analyse whether single items and total scores on the screening instrument (Suicide risk check list, SRC) (Table 1) implemented at the psychiatric acute ward could predict subsequent suicide among all patients admitted to the ward over a six-year period. In addition, we investigated the predictive role of diagnosis, total number of admissions related to suicide risk per patient to the acute ward, and a range of treatment variables.

**Table 1:** Suicide Risk Check list (SRC).

*Part A General questions*
1. Presence of mental disorder
2. Previous suicide attempts (excluding self-harm with no suicidal attempt)
3. Suicide in the family
4. Alcohol or drug dependence
5. Disruption of important relations
6. Loss of self-esteem/ defamation
7. Serious somatic illness
*Part B Specific questions*
1. Presence of suicidal thoughts/ thoughts about suicide
2. Are the thoughts present continuously/ on and off
3. The patient has a suicide plan, including method and circumstances
4. Presence of voices that the patient should complete suicide
5. Thoughts about death and wishing to be dead
6. Reduced impulse control
7. Lack of social network
8. Feelings of hopelessness, indifference, and/ or aggression
9. Lack of protecting factors (e.g. children, family, boyfriend/ girlfriend, animals, religion, hobby)
10. Other recently occurred issues relevant to suicide risk (e.g. work related issues, living conditions)

Each item is answered as no/ absent (= 0), possibly/ moderately present (= 1), yes/ present (= 2), or do not know/ information is missing (= 0)

## Material and methods

In this prospective case-control study, all patients admitted to a locked-door psychiatric acute ward at Blakstad hospital, Vestre Viken hospital trust, in the period January 1 2008 to December 31 2013 were identified by extracting information from the electronic patient journal (EPJ) system DIPS. The acute ward consisted of two subunits with together 24 inpatient beds and covers a catchment area for 170,000 community inhabitants.

In Norway, all citizens are covered by government-funded health services, including psychiatric treatment, independent of social background and status. A central principle for specialized psychiatric health care is that patients are to receive treatment at the lowest effective level of care. Accordingly, patients who are admitted to locked-door psychiatric acute wards represent a highly selected group, being characterized by a severe clinical condition, typically with the need of acute treatment and protection, often with imminent danger to one self or others.

### Participants

A total of 1976 patients had been admitted to the acute ward during the study period, 983 women (49.7%) and 993 men (50.3%). In this period, approximately 40% of all admissions were coerced/ involuntary, and median length of stay was 10 days. We provided the social security numbers of the 1976 patients to the National Institute of Public health (NIPH), which matched the patients to registered deaths in the NCDR by December 2014, as evidenced in a report from NCDR of December 2015. The matching procedure led to the identification of 246 deaths among the 1976 patients (12.4%), of which ICD-10 diagnosis for cause of death was stated for 219 patients. Forty patients (18.3% of all deaths; 2.0% of the overall population at the acute ward), had been entered with an ICD-10 code for suicide (X61-X81). This group consisted of 18 women and 22 men, with a mean age at death of 47.7 years (SD = 15.0).

We established a control group three times the size of the suicide group (54 females and 66 males) in order to increase the power of the study. First, using a random number generator, a list of 300 patients was generated from the 1730 patients in the baseline group who were not registered as dead. For each of the 40 patients in the suicide group, the first patient on this list with the same gender and year of birth was included in the control group. When later inspecting the EPJs using a check list (see below), patients in the control group who lacked completion of the SRC (*n* = 14) were removed and replaced with another patient of the same gender and year of birth. At the time point for their last admission to the psychiatric acute ward in the 2008–2013 period, which on average was autumn 2010, the patients’ mean age in the control group was 46.4 years (SD = 13.7), compared to 46.7 years (SD = 14.6) in the group of suicide completers. Of the 160 patients included in the two study groups, 133 were ethnic Norwegians, 15 were from other European countries, seven from Asian countries, and five from African countries or South America.

Inspection of the EPJs revealed that the SRC was not available for four suicide completers, leaving 36 of these patients with SRC data, 18 female and 18 male patients (mean age at last admission, 45.6 years, SD = 13.9).

### Measures

#### Suicide Risk Check list (SRC)

The SRC consists of 17 items that operationalize risk factors recommended by the Norwegian Health Directorate [20] (Table 1) and consists of two parts. Part A focuses on general risk factors and Part B on more specific or current themes (Table 1). Each item is scored on a three-point Likert scale, where 0 = no/ absent, 1 = possibly/ moderately present, and 2 = yes/ present. If insufficient information is available to score an item, this is entered as “do not know”, which we equalled to a zero score. The SRC was administered by medical doctors in the admission meeting, typically within two hours from admission to the acute ward. All involved medical doctors had been instructed by other personnel on how to use the SRC. However, no systematic training plan was implemented at the ward, and instrument administration may have varied among clinicians. For patients with multiple admittances during the study period, SRC was extracted for the first and last admittance.

In a partial validation study, we administered the SRC together with the Suicide assessment scale (SUAS)[21] to a composite group of 33 patients and 33 normal healthy participants (hospital employees) who self-completed the instruments [22]. Linear regression using sub-scores on the two parts of the SRC as independent variables and SUAS total score as dependent variable showed a strong positive association, *F*(2, 64) = 14.1, *p* < .001, with R^2^ at 83%. Each of the two main parts of the SRC contributed significantly to the association with SUAS (*p* < .001).

We developed a check list to extract information from EPJ for the first and last admission for each patient (see Table 2):

**Table 2:** Check list used to inspect electronic patient journals.

1. For the first and last (if more than one) admission to the acute ward at Blakstad hospital in the study period:
a. Gender and ethnicity
b. Primary diagnosis (ICD-10) as set in routine clinical care
c. Observational status for the patient at admission to the acute ward: constant observation, intermittent observation (the patient is checked upon every 5–15 minutes), no observation schedule
d. Type of follow up after discharge: other inpatient treatment, outpatient treatment, community mental health services, and other types of follow up
e. At enrolment and discharge, the use (yes, no) of any psychopharmacological treatments classified by the WHO scheme (http://www.whocc.no/atc_ddd_index/) as N03A antiepileptics, N05A antipsychotics, N05B anxiolytics, N05C hypnotics/ sedatives, and N06A antidepressants
2. Number of stays before and after start of the study period in 2008 at, respectively, inpatient hospital wards, inpatient wards at district psychiatric centers, and inpatient wards for substance abuse treatment
3. All-time number of admissions to the psychiatric acute ward at Blakstad hospital with suicide risk noted as part of the patients’ problems
4. Outpatient treatment (yes, no) for each of the years 2008–2013
5. The use of electroconvulsive treatment in the study period (yes, no)
6. The use of each of the following coercive measures (yes, no) in the study period: coercive hospitalization, coercive medication, coercive use of mechanical constraints (belts)/ short-term holding, and coercive open-area seclusion

### Statistical analysis

In a reliability analysis, for patients with SRCs from both a first and last admission (*n* = 53), scores on the two SRCs were compared using Pearson correlation analysis. In univariable analysis, differences between the suicide group and control group were assessed using t-test for independent samples for normally distributed variables (SRC total and subtotal scores), Mann-Whitney U-test for non-normally distributed continuous variables (SRC items, number of stays at the various inpatient units, number of admissions to the acute ward with suicide problems, years with outpatient treatment), and Chi square test for categorical variables (diagnoses, use of medications, electroconvulsive treatment, use of coercive measures, observational status at admission, type of follow-up after discharge). For groups of variables that included multiple tests (SRC items, diagnostic categories, treatment variables), *P*-values are first given without adjustment for multiple tests and then following Bonferroni corrections.

Contingent on the actual results, we performed a series of logistic regression analysis to test whether associations of treatment variables with suicide in the univariable analyses rather depended on a relation between the treatment variables and differential distribution of diagnoses in the suicide and control groups. Predictors in these analyses were the respective treatment variables and a categorization of the diagnoses, which is further detailed in the result section. We tested the association between time from discharge to suicide and SRC scores in the suicide group using Pearson correlation analysis.

Variables associated with suicide at the *p* < 0.10 level in the univariable analyses (uncorrected), were entered as predictors in a multivariable logistic regression analysis, with suicide as dependent variable. Collinearity was here estimated by inspecting correlations between the independent variables. All statistical analyses were performed in SPSS version 23 [23].

### Ethics statement

The study was approved by Regional Committees for Medical and Health Research Ethics, Health region south-east, Norway, 2014/470 (REC). REC gave dispensation from the requirement of informed consent, based on §35 in the Norwegian law for regulation of research into health issues. Reasons behind this decision included that a central subgroup of the patients were dead, a number of others would be likely to exhibit reduced competence in providing informed consent, and it would have been difficult to reach all relevant patients. Data extraction from the Norwegian Causes of Death Registry (NCDR) was approved by the Norwegian Institute of Public Health, yp/14-0043.

## Results

**Fig 1:**
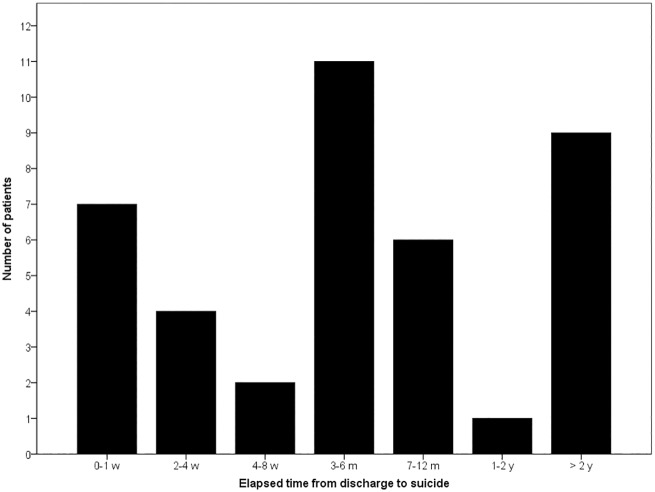
Elapsed time from discharge from the psychiatric acute ward to suicide in 40 patients.

In the group of suicide completers, suicide occurred an average of 345.4 days following discharge from the patients’ last admission to the acute ward (median = 120.5 days, SD = 474.5; Fig 1).

### Comparing scores on the suicide risk scale for first and last admissions

Among the 160 participants, 107 had one admission to the psychiatric acute ward in the study period, while 53 patients had been enrolled twice or more and had two complete SRCs available for the study. For the 53 patients with two SRCs, the total scores of these SRC’s correlated moderately, *r* = 0.40, *p* = 0.003. Moderate correlations also were seen for scores on the first and last admission for SRC part A, *r* = 0.62, *p* < 0.001, and SRS part B, *r* = 0.30, *p* = 0.033. Three items on SRC part A could be expected to be more consistent than other items over time and instrument administrations: previous suicide attempts, previous suicide in the family, and presence of severe somatic disorder. The correlations between the first and last admission for these three items were moderate to strong, *r*’s = 0.71, 0.70 and 0.77, respectively (*p*-values < 0.001).

For previous suicide attempts, 25 out of 27 patients (94.8%) with confirmatory answer at first admission were scored with the same answer at the last admission. Six patients noted a history of suicide in their family at first admission, but only three (50%) were recorded with the same answer at last admission. Hence, reliability appeared to be only moderate and to vary across SRC items.

### Suicide risk scale and suicide

The total score on the SRC was higher in the suicide group than the control group on both the first admission, *M* = 11.7 (SD = 5.2) vs *M* = 9.7 (SD = 5.0), *t*(154) = 2.1, *p* = 0.034, and the last admission, *M* = 11.9 (SD = 5.3) vs *M* = 9.2 (SD = 5.1), *t*(154) = 2.8, *p* = 0.006. For SRC part A and B, subtotal scores were significantly higher in the suicide group than the control group only for part B and only at last admission, *M* = 6.8 (SD = 4.1) vs *M* = 4.7 (SD = 3.9), *t*(154) = 2.7, *p* = 0.008. Trend level associations were reached for part A at last admission *t*(154) = 1,7, *p* = 0.098, and, for the first admission, each of part A, *t*(154) = 1.7, *p* = 0.088, and part B, *t*(154) = 1.8, *p* = 0.080. On this basis, in all subsequent analyses we used the SRC from the last admission only.

Of the seven items in SRC part A, only item 2, previous suicide attempts, was scored significantly higher in the suicide group (*p* = 0.019). Of the 10 items in SRC part B, five were significantly higher in the suicide group (*p*-values ≤ 0.01); thoughts about suicide, continuity of suicidal thoughts, plans about suicide, thinks a lot about death/ wishes to be dead, and feelings of hopelessness, indifference or aggression. When Bonferroni corrected for multiple testing (*p* = 0.05/ 17 tests = 0.00294), only two SRC items remained significant: presence of suicidal thoughts and wishing to be dead (Table 3)

**Table 3:** Associations of items in the Suicide Risk Check list (SRC) with suicide.

SRC item	Suicide group *M* (SD)	Control group *M* (SD)	*P* value^1^
A1 Presence of mental disorder	1.53 (0.65)	1.55 (0.65)	0.83
A2 Previous suicide attempt	1.11 (0.98)	0.68 (0.89)	0.019
A3 Suicide in the family	0.25 (0.65)	0.25 (0.65)	0.95
A4 Alcohol/ drug dependence	0.72 (0.85)	0.60 (0.80)	0.42
A5 Disruption of important relation	0.72 (0.91)	0.53 (0.77)	0.30
A6 Loss of self-esteem/ defamation	0.53 (0.70)	0.57 (0.72)	0.81
A7 Serious somatic illness	0.31 (0.62)	0.30 (0.64)	0.83
B1 Presence of suicidal thoughts	1.14 (0.87)	0.63 (0.84)	0.002*
B2 Continuity of suicidal thoughts	0.81 (0.86)	0.40 (0.64)	0.007
B3 Has a suicide plan	0.61 (0.90)	0.29 (0.65)	0.038
B4 Hearing voices of committing suicide	0.06 (0.33)	0.13 (0.47)	0.26
B5 Wishes to be dead	0.86 (0.83)	0.43 (0.71)	0.002*
B6 Reduced impulse control	0.75 (0.69)	0.63 (0.75)	0.26
B7 lack of social network	0.50 (0.70)	0.61 (0.76)	0.48
B8 Hopelessness, indifference, aggression	1.17 (0.77)	0.78 (0.77)	0.010
B9 Lack of protecting factors	0.39 (0.60)	0.38 (0.62)	0.78
B10 Other recent, relevant issues	0.47 (0.70)	0.46 (0.71)	0.83

^1^Mann-Whitney U-test, *p*-values are uncorrected for multiple tests.

*Reached Bonferroni-adjusted *p*-value for 17 tests at *p* = 0.00294.

We extracted the six SRC items that were higher in the suicide group (without Bonferroni correction) into a new scale, “SRC brief”, with total scores ranging from 0–12. The mean score on this scale was 78.1% higher in the suicide group (*M* = 5.7, SD = 4.1) than in the control group (*M* = 3.2, SD = 3.2), *t*(154) = 3.8, *p* < 0.001. For the remaining 11 items on SRC considered together (SRC total scores minus SRC brief), no association was seen with suicide, *t*(154) = 0.4, *p* = 0.67.

For the group of suicide completers, no association was evident for elapsed time from discharge to suicide with the total score on the original SRC, *r* = -0.09, *p* = 0.62, or with the total score on the SRC brief scale, *r* = -0.14, *p* = 0.41.

### Number of admissions with suicide problems

In the suicide group, 34 out of 40 patients (85%) had at least one admission (all time) to the acute ward with suicide problems, compared to 70 out of 120 patients (58%) in the control group, χ^2^(1, 159) = 9.4, *p* = 0.002. The number of such admissions per patient also was higher in the suicide group (*M* = 1.8, SD = 2.1, range 0–10) than in the control group (*M* = 1.0, SD = 1.6, range 0–13), *p* = 0.003.

### Diagnoses

We sorted primary diagnoses into eight categories (Table 4). Among these diagnostic categories, only Depressive disorder (F32-33) was more prevalent in the suicide group, χ^2^(1, 159) = 4.2, *p* = 0.041 (uncorrected for multiple tests), whereas schizophrenia (F20.0–9) tended to be more prevalent in the control group χ^2^(1, 159) = 3.7, *p* = 0.054. When adjusting for multiple tests (*p* = 0.05/ 8 tests = 0.00625), none of the diagnostic categories were significantly different in the two groups.

**Table 4:** Primary diagnoses at last admission to the acute ward.

Diagnostic group	Suicide group (*n*, %)	Control group (*n*, %)	*P* value^1^
F10-19 Disorders associated with substance use	6 (15.0%)	16 (13.3%)	0.79
F20 Schizophrenia	1 (2.5%)	16 (13.3%)	0.054
F21-29 Other psychosis	3 (7.5%)	9 (7.5%)	1.0
F31 Bipolar disorder	3 (7.5%)	15 (12.5%)	0.39
F32-33 Depressive disorder	16 (40.0%)	28 (23.3%)	0.041
F40-48 Neurotic/ stress related disorders	3 (7.5%)	15 (12.5%)	0.39
F60-69 Personality disorders	2 (5.0%)	7 (5.8%)	0.84
Other diagnoses/ unspecified/ missing	6 (15.0%)	14 (11.7%)	0.58
All diagnoses	40 (100%)	120 (100%)	-

^1^Chi square tests, *p*-values are uncorrected for multiple tests. No diagnostic category was significantly different in the two study groups when adjusting for multiple testing (Bonferroni, *p* = 0.00625).

Based on the above, we reduced diagnostic group from eight to the following three, and which we used in subsequent regression analyses: Depression, Schizophrenia and Other diagnosis.

### Treatment variables

The associations with suicide for number of admissions to inpatient wards and number of years with outpatient treatment are summarized in Table 5. None of these variables were associated with suicide even before adjustment for multiple tests.

**Table 5:** Associations of inpatient stays and outpatient treatment with suicide.

Type of treatment	Suicide group (*M*, SD)	Control group (*M*, SD)	*P* value^1^
*Number of stays at inpatient wards*:			
Hospital wards, before start of study period^2^	1.2 (3.1)	2.1 (7.4)	0.70
Hospital wards, after start of study period	2.4 (2.5)	2.2 (2.3)	0.69
DPC wards, before start of study period	0.32 (1.5)	0.22 (0.8)	0.99
DPC wards, after start of study period	0.7 (1.3)	1.5 (4.6)	0.62
Wards for alcohol/ drug addiction, before start of study period	0.0 (0)	0.01 (0.09)	0.56
Wards for alcohol/ drug addiction after start of study period	0.0 (0)	0.18 (0.9)	0.12
All inpatient wards, total, before start of study period	1.6 (4.2)	2.3 (7.6)	0.75
All inpatient wards, total, after start of study period	3.1 (3.2)	3.9 (5.5)	0.42
All inpatient wards, all time periods	4.6 (5.9)	6.2 (11.7)	0.48
*Number of years with outpatient treatment in the study period*	1.5 (1.3)	1.9 (1.8)	0.53

^1^Mann-Whitney U-test, *p*-values are uncorrected for multiple tests.

^2^Start of study period was January 2008. DPC—District psychiatric center.

The associations of other treatment variables with suicide are depicted in Table 6. Significant associations (uncorrected) with suicide status were seen for the coercive use of mechanical constraints, χ^2^(1, 159) = 4.7, *p* = 0.030, which was more frequent in the control group; the use of antipsychotics at intake, χ^2^(1, 159) = 4.7, *p* = 0.030 (more frequent in the control group), antidepressants at intake, χ^2^(1, 159) = 4.4, *p* = 0.035 (more frequent in the suicide group), and antipsychotics at discharge, χ^2^(1, 159) = 4.9, *p* = 0.026 (more frequent in the control group), with a similar trend for antidepressants at discharge χ^2^(1, 159) = 2.7, *p* = 0.098 (more frequent in the suicide group). No association remained significant after Bonferroni correction for multiple tests.

**Table 6:** Associations of treatment variables with suicide.

Treatment variable	Suicide group	Control group	*P* value^1^
Observational status at admission (*n*)	Constant: 2	Constant: 6	0.78
	Intermittent: 19	Intermittent: 56	
	None: 15	None: 58	
Type of follow up after discharge (*n*)	Inpatient: 10	Inpatient: 37	0.18
	Outpatient: 10	Outpatient: 53	
	Other: 13	Other: 29	
N03A antiepileptics at admission (%)	25.7%	21.9%	0.63
N05A antipsychotics at admission (%)	25.7%	46.2%	0.030
N05B anxiolytics at admission (%)	28.6%	26.9%	0.84
N05C hypnotics/ sedatives at admission (%)	25.7%	24.4%	0.87
N06A antidepressants at admission (%)	51.4%	31.9%	0.035
N03A antiepileptics at discharge (%)	28.1%	20.0%	0.32
N05A antipsychotics at discharge (%)	31.3%	53.3%	0.026
N05B anxiolytics at discharge (%)	21.9%	25.8%	0.65
N05C hypnotics/ sedatives at discharge (%)	28.1%	24.2%	0.65
N06A antidepressants at discharge (%)	53.1%	37.0%	0.098
Electroconvulsive treatment	7.5%	6.7%	0.86
Coercive admittance under the Norwegian Mental Health Act (%)	47.5%	47.5%	1.0
Coercive use of medication (%)	5.0%	10.0%	0.33
Coercive use of mechanical constraints (%)	0%	10.8%	0.030
Coercive use of open-area seclusion (%)	2.5%	7.5%	0.26

^1^Chi square tests, *p*-values are uncorrected for multiple tests.

We tested whether the association with suicide status for the noted significant treatment variables reflected the different prevalence of depressive disorders and schizophrenia in the two study groups, using a series of logistic regression analyses. In these analyses, suicide was no longer associated with any of the treatment variables, *p*-values > 0.12, indicating that their association with suicide occurred indirectly through an association with diagnosis.

### Multivariable analyses

Based on the univariable analyses, in a final logistic regression analysis, we used, as independent variables, diagnostic group (schizophrenia, depression, other diagnosis), the SRC brief scale with a 0–12 range, and number of admissions to the acute ward where suicide issues were part of the problem. In this model, only SRC brief had a significant effect upon suicide status, *β* = 0.16, OR = 1.18 (95% CI = 1.05–1.32), *p* = 0.004, with no effects of diagnosis (*p* = 0.16) or admissions with suicide problems (*p* = 0.19). Explained variance (Nagelkerke *R*^*2*^) was 17.0%. The correlations between pairs of independent variables all fell below |*r*| < 0.22, indicating no significant collinearity problem.

Positive predictive power of the SRC brief scale was limited when considering all 1976 patients enrolled to the acute ward, estimated to be 1 in 31 for a score of 6, 1 in 17 for scores of both 8 and 10, and 1 in 5 for a maximum score of 12. Moreover, even a cut-off score of 12 would miss 86% of the suicide completers.

We reran the regression analysis by extending the SRC brief scale with an item for admissions to the acute ward with suicidal problems (0 for none, 1 for one and 2 for more than one) and with an item for diagnosis, with schizophrenia entered as 0, other diagnosis as 1, and depression as 2. Using this extended SRC brief scale (range 0–16) as predictor of suicide in logistic regression, the association was significant at *p* < 0.001, but with a marginally increased odds ratio as compared to the SRC brief alone, OR = 1.21 (95% CI = 1.10–1.33). Explained variance (Nagelkerke *R*^*2*^) was 14.9%, less than for the SRC brief scale without the extensions.

## Discussion

We used a prospective case-control design to investigate suicide risk factors in the general patient population (*n* = 1976) admitted to a locked-door psychiatric acute ward over a six year period. Two items on the SRC, a risk instrument based on national guidelines, were given significantly higher scores in patients who later completed suicide compared to a matched control group, when adjusting for multiple testing. These items assessed presence of suicidal thoughts and the patient wishing to be dead. Four more SRC items were significant in unadjusted tests: previous suicide attempts, the continuity of suicidal thoughts, the patient having a suicide plan, and feelings of hopelessness, indifference, or aggression. Considered together, these six items could only weakly predict suicide. We found no associations with suicide for alcohol and drug dependence, suicide in the family, disruption of important relations, serious somatic illness, commanding voices of suicide, lack of social network, or reduced impulse control. Depression and number of admissions with suicide problems showed trend associations with suicide in univariable analyses but not in multivariable logistic regression.

We are aware of no previous study on predictors of suicide in the general population of patients admitted to psychiatric acute wards. Prior research instead have focused on selected patient groups admitted to acute wards, in particular those with previous suicide attempts, on patients admitted to other types of inpatient wards, on the population at large using registers that included information of hospitalization, or on other outcome measures than suicide such as self-harm, suicide attempts, and suicidal thoughts. However, case-control studies of the overall population of patients treated at other types of inpatient wards have reported findings comparable to ours. In these studies, the most common predictors of suicide were previous incidents of self-harm and suicide attempts, suicidal ideation, feelings of hopelessness, and depression, with no or inconsistent effects of factors such as substance abuse, somatic illness, and compulsory care [9,11,24–26]. This overlap in findings suggests that factors that predict suicide are similar for patient populations at different types of inpatient wards.

Even if scores on items for suicide-specific ideations at admission to the psychiatric acute ward were associated with suicide in our participant group, these items combined had a low precision in predicting suicide. This is consistent with all prior research on groups of psychiatric patients and it questions the utility of deploying clinical resources to assess suicide risk [6]. A general conclusion from research on suicide risk prediction is the lack of any items or information that to a useful degree permit the identification of persons who will complete suicide [27,28]. Central to this is the low rate of suicide in any population, which severely limits the possibility to predict this type of event [27]. Other complicating factors are that most subjects who complete suicide do so in the first or second attempt, limiting the predictive role of prior suicide attempts, and the majority has never been evaluated at a psychiatric ward [29].

Due to the limited precision of risk categorization, several authors argue for a shift from risk categorization towards a focus on prevention and intervention strategies, including increased engagement with the individual patient, their specific problems and circumstances [30–32]. Towards this end, our findings support the in-depth assessment of suicidal ideation since it de facto is associated with subsequent suicide. Ways to do this are available, such as by using the Suicide Status Form (SSF) where suicide specific issues are addressed in detail in a collaborative manner between the patient and clinician [33–36].

### Limitations

The ability to identify associations with suicide in this study may have been limited by a probably constrained reliability of the ordinary-care screening procedure for suicide risk. Also the reliability of diagnoses is unclear since they were set in ordinary clinical care. Moreover, the relatively few suicides that were included may have constrained the ability to identify true associations with risk factors. At the same time, the (weak) associations that were identified with completed suicide are consistent with prior research on other patient groups and settings. Strengths include the focus on the general population of patients admitted to a psychiatric acute ward, and the utilization of data from routine suicide risk screening in ordinary care, which increase the utility of the findings for clinicians at psychiatric acute wards. It should be stressed that the findings from the present study are not possible to generalize to the general population.

## Supporting information

S1 Dataset Fosse et al minimal data set. (SAV)
